# Frailty and the confidence to plan ahead: Decision-making self-efficacy and advance care planning among older adults receiving home healthcare

**DOI:** 10.1017/S1478951526102636

**Published:** 2026-05-21

**Authors:** Chi Hsien Huang, Cheng-Pei Lin, Kelly Yen-Chih Chen, Jung-Yu Liao, Shao-Yi Cheng, Wei-Zhe Tseng, Hiroyuki Umegaki, Hsien-Cheng Chang, Chia-Ming Li, Wen-Jung Sun, Hung-Yi Chiou, Sang-Ju Yu, Chao A. Hsiung, Ping-Jen Chen

**Affiliations:** 1 Department of Family Medicine and Community Medicine, E-Da Hospital, I-Shou University, Kaohsiung, Taiwan; 2 College of Medicine, I-Shou University, Kaohsiung, Taiwan; 3 College of Nursing, Kaohsiung Medical University, Kaohsiung, Taiwan; 4 Institute of Community Health Care, College of Nursing, National Yang Ming Chiao Tung University, Taipei, Taiwan; 5 Cicely Saunders Institute, Florence Nightingale Faculty of Nursing, Midwifery and Palliative Care, King’s College London, London, UK; 6 Department of Health Promotion and Health Education, National Taiwan Normal University, Taipei, Taiwan; 7 Department of Family Medicine, College of Medicine and Hospital, National Taiwan University, Taipei, Taiwan; 8 Department of Family Medicine and Division of Geriatrics and Gerontology, Kaohsiung Medical University Hospital, Kaohsiung Medical University, Kaohsiung, Taiwan; 9 Department of Public Health, College of Medicine, National Cheng Kung University, Tainan, Taiwan; 10 Department of Community Healthcare & Geriatrics, Nagoya University Graduate School of Medicine, Nagoya, Japan; 11 Office of the Commissioner, Keelung City Health Bureau, Keelung, Taiwan; 12 Department of Family Medicine, Lotung Poh-Ai Hospital, Yilan, Taiwan; 13 Family Medicine Department, National Taiwan University Hospital Beihu Branch, Taipei, Taiwan; 14 Department of Family Medicine, Tainan Municipal Hospital (Managed by Show Chwan Medical Care Corporation), Tainan, Taiwan; 15 Department of Community Medicine, Taipei City Hospital, Taipei, Taiwan; 16 Institute of Population Health Sciences, National Health Research Institutes, Miaoli, Taiwan; 17 Home Clinic Dulan, Taitung, Taiwan; 18 National Center for Geriatrics and Welfare Research, National Health Research Institutes, Miaoli, Taiwan; 19 School of Medicine, College of Medicine, National Sun Yat-sen University, Kaohsiung, Taiwan

**Keywords:** Frailty, advance care planning, self-efficacy, home healthcare, decision-making

## Abstract

**Objectives:**

The relationship between frailty, self-efficacy, and advance care planning (ACP) remains unclear in Asia. This study examined how frailty status relates to decisional self-efficacy, ACP engagement, and advance directive completion among older adults receiving home healthcare in Taiwan.

**Methods:**

A cross-sectional analysis was conducted using baseline data from a nationwide cohort in Taiwan. Participants (*N* = 358) were categorized by Clinical Frailty Scale (CFS): mildly frail (CFS 4–5, *n* = 60), moderately frail (CFS 6, *n* = 83), severely frail (CFS 7, *n* = 147), and very severely frail (CFS 8–9, *n* = 68). ACP engagement and decision-making self-efficacy were assessed using Likert scales.

**Results:**

Patients with greater frailty had lower odds of high decisional self-efficacy (CFS: 8–9: odds ratio [OR] = 0.38, 95% confidence interval [CI] = 0.14–1.07) but higher odds of ACP engagement (CFS: 6: OR = 3.38, 95% CI = 1.40–8.17; CFS: 7: OR = 2.52, 95% CI = 1.08–5.89) compared with mildly frail individuals. However, this increase did not extend linearly to the very severely frail group. Advance directive completion remained low across all frailty levels (4.8–10.0%) and was not significantly associated with frailty status.

**Conclusions:**

Frailty was associated with lower decisional self-efficacy but higher readiness for ACP, revealing a divergence between perceived confidence and planning motivation. Despite greater engagement, advance directive completion remained low. Stage-sensitive, values-based approaches may help bridge the gap between intention and documentation across the frailty spectrum.

## Introduction

Home healthcare (HHC) refers to healthcare services that provide longitudinal interdisciplinary care to homebound or functionally impaired patients who have difficulty accessing traditional outpatient clinics (Ritchie et al. 2018). These services can vary widely geographically and internationally, encompassing diverse programs and models. The scope of services ranges from acute hospital care at home to long-term support and treatment for chronic or terminal illnesses. The primary goal is to deliver integrated, optimal care to patients in their familiar home environments (Liao et al. 2020).

Patients receiving HHC often exhibit higher levels of frailty, disability, and malnutrition (Kojima et al. 2018; Huang et al. 2019; Proietti and Cesari 2020). For instance, a Norwegian study revealed that 76% of older HHC patients had moderate or severe frailty, with 67% of this group dying within a 2-year follow-up period (Krogseth et al. 2021). Given the increased vulnerability of individuals with frailty to poor health outcomes and adverse events (Soones et al. 2017; Ritchie and Leff 2018), advance care planning (ACP) is a critical aspect of care for this population (Crooms and Gelfman 2020).

ACP is a process facilitating the discussion and understanding of personal values, life goals, future medical choices, and end-of-life care preferences (Sudore et al. 2017). It is important to distinguish between ACP engagement, which represents the relational and psychological process of readiness and communication, and advance directive (AD) completion, which is the formal, often legal, outcome of that process. While effective communication regarding ACP enhances the ability of older people to participate in shared decision-making, “readiness” to engage in these discussions does not always result in the immediate completion of formal documentation (Houben et al. 2014; Kinley and Flemming 2021).

Decision-making self-efficacy, or an individual’s perceived ability to engage in discussions and make one’s own decisions, emerges as a crucial factor for ACP and AD (Farley 2020). However, people with frailty frequently manifest diminished self-efficacy, further compounded by age-related multimorbidity and functional dependence (Cybulski et al. 2017; Whitehall et al. 2021). Furthermore, communication barriers, including cognitive and physical decline related to frailty, may hinder individuals’ ability to actively engage in decision-making processes (Doba et al. 2016; Maruta et al. 2022).

In Asian countries, cultural factors such as collective decision-making and misperceptions of frailty as a total loss of capacity – may influence the decisional self-efficacy among older people (Martina et al. 2021b). Health literacy, patients’ knowledge of their disease, and ACP may also pose challenges in facilitating ACP discussions (de Vries et al. 2019; Martina et al. 2021a). In HHC settings, family dynamics, social support, and the availability of healthcare resources also play pivotal roles in shaping the effectiveness of ACP among frail patients (Boerner et al. 2013; Martina et al. 2021a). However, little is known about the relationship between frailty and ACP in patients receiving HHC. This study aims to investigate the association between levels of frailty, decision-making self-efficacy, ACP engagement, and AD completion among frail older people receiving HHC in a nationwide cohort in Taiwan.

## Methods

This study is part of the HOme-based Longitudinal Investigation of the multidiSciplinary Team Integrated Care (HOLISTIC) study, which is the first nationwide prospective longitudinal study of HHC in Taiwan (Liao et al. 2020). The HOLISTIC study involved a 2-year observation period starting in November 2019, covering 18 sites nationwide. It included 6 assessments of patients receiving HHC services and their caregivers. Detailed recruitment information is available in our published article (Liao et al. 2020). For the present study, we have utilized the baseline patient data from the HOLISTIC study, including age, sex, Charlson Comorbidity Index (CCI), education, marital status, living status, religious beliefs, and income.

### Participants

In the HOLISTIC study, patients were considered eligible if they met the following inclusion criteria: (1) age over 50 years, (2) receipt of HHC for at least 2 months, (3) Barthel index <60, (4) ability to communicate with the interviewer in a mutually understood language, and (5) cognitive impairment, if present, was compensated for by a cognitively competent caregiver who facilitated communication with the interviewer. In the HOLISTIC protocol, caregiver facilitation was employed primarily to support patients with cognitive impairment and to help interviewers’ understanding of patients’ pre-dementia values and preferences, rather than to act as proxy decision-makers. The interviews were specifically designed to prioritize patients’ perspectives, utilizing a first-person approach to reflect their personal experiences while promoting inclusivity for vulnerable populations. This approach aligns with the concept of supported decision-making, ensuring accurate representation of the patients’ perspectives through those who know them best and can advocate for them.

During the initial recruitment phase of the HOLISTIC study, HHC team members provided an overview of the study protocol. To ensure effective communication, interviewers received training that included standardized explanations and education on ACP, enabling them to effectively address any questions or misconceptions. These trained interviewers then contacted participants by telephone to schedule home visits, where they offered a more detailed explanation of the study’s purpose and procedures. After confirming participants’ willingness and eligibility, written informed consent was obtained, and interviews were conducted. The caregivers or family members, who knew the patients well and could best advocate for them, facilitated the interviews, ensuring accurate representation of the patients’ perspectives.

### Measures

Within the HOLISTIC study, a structured questionnaire consisting of 5 domains (health-related outcomes, end-of-life issues, continuity and coordination of care, care resource utilization and costs, and caregiving burden) was used (Liao et al. 2020). For the purpose of this study, only measures relevant to our research objectives are discussed, including the 9-point Clinical Frailty Scale (CFS), the DEcision-making Participation Self-efficacy scale (DEPS), ACP as measured by the 4 questions in ACP Engagement Survey, and demographic information.

A trained nurse conducted a comprehensive geriatric assessment, evaluating frailty using the 9-point CFS. This scale offers a distinct description for each score, ranging from 1 (very fit) to 9 (terminally ill), and is internationally recognized as a common tool for evaluating frailty among older individuals (Church et al. 2020). The translated CFS has shown strong validity and reliability in the Taiwanese population (Chou et al. 2022). In this study, we classified patients with CFS scores of 4–5 as mildly frail, 6 as moderately frail, 7 as severely frail, and 8–9 as very severely frail. We included patients aged 65 and older, excluding those with CFS scores of 1–3 (*n* = 25), as they were not considered frail.

The DEPS was used to assess patients’ confidence in their participation in shared decision-making (Arora et al. 2009). The scale consists of 5 questions, with patients responding on a 5-point Likert scale ranging from 1 (not at all confident) to 5 (completely confident). Higher scores indicate greater perceived self-efficacy in engaging with treatment-related decisions.

A 4-item ACP Engagement Survey was used to assess patients’ readiness to engage in discussion concerning their personal beliefs and future treatment preferences with both family members and healthcare teams (Sudore et al. 2017). Patients responded on a 5-point Likert scale, ranging from 1 (never thought about it) to 5 (already completed). Higher scores indicate greater preparedness in understanding and sharing personal values, life goals, and preferences for future medical care. The reliability and validity of the ACP Engagement Survey in Taiwan have been verified, demonstrating good internal consistency (Wei et al. 2022).

To ensure clinical relevance and reflect the distribution within our cohort, outcome variables were categorized as tertiles: DEPS scores (≤9, 10–16, and >16) and ACP scores (4, 5–6, and >6). In both measures, higher scores indicate superior outcomes – specifically, greater perceived self-efficacy in decision-making and higher levels of ACP preparedness, respectively. Covariates encompassed demographic variables, including age, sex, comorbidity status as determined by the CCI (Charlson et al. 1994), educational level, marital status, living arrangements, religious affiliation, monthly income, and the presence of indwelling tubes or catheters. CCI was ascertained based on past and current medical records.

### Statistical analysis

A multinomial logistic regression was used to analyze the association between CFS scores and outcome variables. Odds ratios (ORs) with 95% confidence intervals (CIs) were calculated after adjusting for all covariates. In the sensitivity analysis, only individuals with a CFS score of 4–5 were included in the mildly frail group to confirm the robustness of our findings. A significance threshold of *p* < 0.05 was utilized to delineate statistical significance across all analyses. Statistical computations were conducted using SAS software, version 9.4 (SAS Institute, Inc., Cary, NC, USA).

## Results

Table 1 offers a comprehensive overview of the characteristics of the study’s patients based on their CFS score. A total of 358 patients (206 women) were enrolled. The study cohort encompassed 60 mildly frail patients (CFS score 4–5), 83 moderately frail patients (CFS score 6), 147 severely frail patients (CFS score 7), and 68 very severely frail patients (CFS score 8–9). The mean age exhibited a progressive increase in tandem with rising CFS scores, ranging from 82 years for mildly frail patients to 86 years for very severely frail patients. CFS scores demonstrated significant disparities in the distribution of age across 3 age categories (<75, 75–84, and ≥85) (*p* < 0.01). Patients with higher CFS scores (8–9) had elevated CCI scores (mean = 3) and were more likely to live with immigrant caregivers and have household incomes exceeding NT$20,001. Additionally, higher CFS scores were significantly associated with the use of indwelling medical devices (*p* < 0.01). While 51.7% of the mildly frail group were able to complete the assessment independently, this proportion decreased to 30.1% for the moderately frail, 9.5% for the severely frail, and 0% for the very severely frail group (*p* < 0.001).

**Table 1.** Characteristics of the study participants by Clinical Frailty Scale (CFS) score Table 1 long description.

	Clinical Frailty Scale score				
**Characteristics**^a^	4−5, *n* = 60	6, *n* = 83	7, *n* = 147	8–9, *n* = 68	*p*-Value
Age, median (Q1–Q3), *n* (%)^b^	82.0 (75.0–87.0)	85.0 (78.0–90.0)	85.0 (79.0–90.0)	86.0 (81.0–92.0)	0.0051
<75	14 (23.3)	9 (10.8)	22 (15.0)	5 (7.4)	0.0896
75−84	23 (38.3)	32 (38.6)	49 (33.3)	21 (30.9)	
≥85	23 (38.3)	42 (50.6)	76 (51.7)	42 (61.8)	
Sex (female), *n* (%)	28 (46.7)	47 (56.6)	89 (60.5)	42 (61.8)	0.2645
CCI, median (Q1–Q3)^b^	2.0 (1.0–3.0)	3.0 (2.0–5.0)	3.0 (2.0–5.0)	3.0 (2.0–5.0)	0.0081
Education (y), *n* (%)					0.0224
Illiterate	14 (21.2)	23 (27.7)	26 (17.7)	14 (20.6)	
≤6	25 (37.9)	49 (59.0)	75 (51.0)	26 (38.2)	
7−12	11 (18.3)	6 (7.2)	27 (18.4)	13 (19.1)	
>12	10 (16.7)	5 (6.0)	19 (12.9)	15 (22.1)	
Marital status, *n* (%)					0.2087
Married	31 (51.7)	43 (51.8)	68 (46.3)	30 (44.1)	
Widowed	24 (40.0)	39 (47.0)	67 (45.6)	36 (52.9)	
Divorced/single	5 (8.3)	1 (1.2)	12 (8.2)	2 (2.9)	
Living status, *n* (%)					<0.0001
Living alone	6 (10.0)	0 (0.0)	1 (0.7)	0 (0.0)	
Only with immigrant carer	1 (1.7)	5 (6.0)	11 (7.5)	5 (7.4)	
Only with family/friend	42 (70.0)	46 (55.4)	58 (39.5)	20 (29.4)	
With immigrant carer/family/friend	11 (18.3)	32 (38.6)	77 (52.4)	43 (63.2)	
Religious beliefs, *n* (%)					0.6637
None	11 (18.3)	15 (18.1)	35 (23.8)	17 (25.0)	
Buddhism/Taoism/I Kuan Tao	40 (66.7)	61 (73.5)	97 (66.0)	46 (67.7)	
Catholicism/Christianity	9 (15.0)	7 (8.4)	15 (10.2)	5 (7.4)	
Income (NTD/month), *n* (%)					0.0009
≤20,000 (with low-income subsidy)	3 (5.0)	2 (2.4)	5 (3.4)	0 (0.0)	
≤20,000 (with low-to-middle income subsidy)	27 (45.0)	28 (33.7)	33 (22.5)	11 (16.2)	
20,001–60,000	24 (40.0)	44 (53.0)	85 (57.8)	37 (54.4)	
>60,000	6 (10.0)	9 (10.8)	24 (16.3)	20 (29.4)	
Indwelling tube or catheter (yes), *n* (%)	5 (8.3)	19 (22.9)	72 (49.0)	54 (79.4)	<0.0001
Urinary catheter (yes), *n* (%)	3 (5.0)	10 (12.1)	32 (21.8)	24 (35.3)	<0.0001
Nasogastric tube (yes), *n* (%)	2 (2.2)	8 (9.6)	47 (32.0)	48 (70.6)	<0.0001
Tracheostomy tube (yes), *n* (%)	0 (0.0)	0 (0.0)	3 (1.9)	4 (5.9)	0.0550
Questionnaire respondent, *n* (%)					<0.0001
Patients	31 (51.7)	25 (30.1)	14 (9.5)	0 (0.0)	
Family/caregivers	29 (48.3)	58 (69.9)	133 (90.5)	68 (100.0)	

CCI = Charlson Comorbidity Index, HBMC = home-based medical care.

a Data were presented as means and standard deviations.

b The Kruskal–Wallis test was used because the data were not normally distributed. The results are presented as medians and interquartile ranges (Q1–Q3).

DEPS scores were significantly higher in mildly frail patients compared to moderately frail patients, severely frail patients, or very severely frail patients (*p* = 0.03) (Table 2). Among mildly frail patients, 25 (41.7%) exhibited DEPS scores exceeding 16, while the corresponding figures for moderately frail, severely frail, and very severely frail patients were 28 (33.7%), 43 (29.3%), and 16 (23.5%), respectively. Notably, CFS scores showed no substantial correlation with responses to the individual 5 DEPS questions. In the multinomial logistic regression analysis, CFS scores exhibited a trend of negative association with DEPS scores. In comparison to mildly frail patients, very severely frail patients (OR = 0.38, 95% CI = 0.14–1.07) exhibited diminished odds of attaining DEPS scores surpassing 16.

**Table 2.** Association between CFS and DEcision-making Participation Self-efficacy scale (DEPS) scores Table 2 long description.

	Clinical Frailty Scale score				
	4–5, *n* = 60	6, *n* = 83	7, *n* = 147	8–9, *n* = 68	*p*-Value
DEPS score, median (Q1–Q3)^a^	15.0 (9.0–23.0)	11.0 (7.0–20.0)	10.0 (6.0–20.0) Missing, *n* = 8; 5.4%	9.0 (5.0–17.0) Missing, *n* = 5; 7.4%	0.0353
≤9, *n* (%)	20 (33.3)	32 (38.6)	67 (45.6)	37 (54.4)	0.0960
10–16, *n* (%)	15 (25.0)	23 (27.7)	29 (19.7)	10 (14.7)	
>16, *n* (%)	25 (41.7)	28 (33.7)	43 (29.3)	16 (23.5)	
Q1. Take part in a detailed discussion with your doctor about the different available options			Missing, *n* = 6; 4.1%	Missing, *n* = 5; 7.4%	0.1512
Very to completely confident	25 (41.7)	27 (32.5)	38 (25.9)	16 (23.5)	
Somewhat to not at all confident	35 (58.3)	56 (67.5)	103 (70.1)	47 (69.1)	
Q2. Let your doctor know if you had any concerns or questions about his or her recommendation			Missing, *n* = 6; 4.1%	Missing, *n* = 5; 7.4%	0.1738
Very to completely confident	25 (41.7)	26 (31.3)	41 (27.9)	15 (22.1)	
Somewhat to not at all confident	35 (58.3)	57 (68.7)	100 (68.0)	48 (70.6)	
Q3. Tell your doctor about the option you would prefer			Missing, *n* = 7; 4.8%	Missing, *n* = 5; 7.4%	0.2063
Very to completely confident	25 (41.7)	26 (31.3)	40 (27.2)	16 (23.5)	
Somewhat to not at all confident	35 (58.3)	57 (68.7)	100 (68.0)	47 (69.1)	
Q4. Work out any differences of opinion with your doctor, should they exist			Missing, *n* = 7; 4.8%	Missing, *n* = 5; 7.4%	0.1872
Very to completely confident	24 (40.0)	26 (31.3)	38 (25.9)	15 (22.1)	
Somewhat to not at all confident	36 (60.0)	57 (68.7)	102 (69.4)	48 (70.6)	
Q5. Take responsibility for making the final decision			Missing, *n* = 7; 4.8%	Missing, *n* = 5; 7.4%	0.8843
Very to completely confident	33 (55.0)	46 (55.4)	74 (50.3)	31 (45.6)	
Somewhat to not at all confident	27 (45.0)	37 (44.6)	66 (44.9)	32 (47.1)	

a The Kruskal–Wallis test was used because the data were not normally distributed. The results are presented as medians and interquartile ranges (Q1–Q3).

Individual and total scores of ACP were not associated with CFS scores (Table 3, panel a). The rates of formal completion for ADs were consistently low across all 4 frailty groups, ranging from 4.8% to 10.0%. Notably, frailty status was not significantly associated with the likelihood of having an AD in place (*p* = 0.44) (Table 3, panel b). Multinomial logistic regression analysis further showed that moderately frail patients (OR = 3.38, 95% CI = 1.4–8.17) and severely frail patients (OR = 2.52, 95% CI = 1.08–5.89) had a higher likelihood of achieving ACP scores exceeding 6 compared to the mildly frail group (Table 4). However, this increase did not follow a linear progression into the very severely frail group (OR = 2.60, 95% CI = 0.96–7.03), suggesting a plateau in engagement as frailty reaches its most advanced stages.

**Table 3.** Association between CFS and (a) UCSF-Advance Care Planning (ACP) scores, and (b) advance directives Table 3 long description.

	Clinical Frailty Scale score				
	4–5, *n* = 60	6, *n* = 83	7, *n* = 147	8–9, *n* = 68	*p*-Value
(a)					
UCSF-ACP scores, median (Q1–Q3)^a^	4.0 (4.0–6.0)	5.0 (4.0–8.0) Missing, *n* = 1; 1.2%	4.0 (4.0–8.0)	4.0 (4.0–8.0)	0.2377
4, *n* (%)	40 (66.7)	40 (48.2)	84 (57.1)	42 (61.8)	0.0724
5–6, *n* (%)	8 (13.3)	9 (10.8)	15 (10.2)	2 (2.9)	
>6, *n* (%)	12 (20.0)	33 (39.8)	48 (32.7)	24 (35.3)	
Q1. How ready are you to talk to your decision maker about the kind of medical care you would want if you were very sick or near the end of life?					0.5708
Definitely planning to do it and already done	9 (15.0)	19 (22.9)	26 (17.7)	15 (22.1)	
Thinking about doing it to never thought	51 (85.0)	64 (77.1)	121 (82.3)	53 (77.8)	
Q2. How ready are you to talk to your doctor about the kind of medical care you would want if you were very sick or near the end of life?					0.6548
Definitely planning to do it and already done	3 (5.0)	9 (10.8)	15 (10.2)	6 (8.8)	
Thinking about doing it to never thought	57 (95.0)	74 (89.2)	132 (89.8)	62 (91.2)	
Q3. How ready are you to sign official papers putting your wishes in writing about the kind of medical care you would want if you were very sick or near the end of life?		Missing, *n* = 1; 1.2%			0.8269
Definitely planning to do it and already done	4 (6.7)	8 (9.6)	11 (7.5)	7 (10.3)	
Thinking about doing it to never thought	56 (93.3)	74 (89.2)	136 (92.4)	61 (89.7)	
Q4. How ready are you to sign official papers naming a person or group of people to make medical decisions for you?		Missing, *n* = 1; 1.2%			0.6007
Definitely planning to do it and already done	1 (1.7)	5 (6.0)	9 (6.1)	3 (4.4)	
Thinking about doing it to never thought	59 (98.3)	77 (92.8)	138 (93.9)	65 (95.6)	
(b)					
Completing advance directives, *n* (%)				Missing, *n* = 1; 1.5%	0.4360
Yes	6 (10.0)	8 (9.6)	7 (4.8)	5 (7.4)	
No	54 (90.0)	75 (90.4)	140 (95.2)	62 (91.2)	

a The Kruskal–Wallis test was used because the data were not normally distributed. The results are presented as medians and interquartile ranges (Q1–Q3).

**Table 4.** Multinomial logistic regression of DEPS scores and ACP scores Table 4 long description.

	DEPS scores^a^ 10–16 vs. ≤9		DEPS scores^a^ >16 vs. ≤9		ACP scores^b^ 5–6 vs. 4		ACP scores^b^ >6 vs. 4	
Characteristic	Full model		Full model		Full model		Full model	
	OR (95% CI)	*p*	OR (95% CI)	*p*	OR (95% CI)	*p*	OR (95% CI)	*p*
Frailty								
4–5	[Reference]		[Reference]		[Reference]		[Reference]	
6	1.00 (0.39–2.60)	0.9993	0.83 (0.35–1.96)	0.6714	1.98 (0.58–6.74)	0.2723	3.38 (1.40–8.17)	0.0069
7	0.64 (0.25–1.60)	0.3363	0.52 (0.23–1.18)	0.1180	1.81 (0.56–5.91)	0.3231	2.52 (1.08–5.89)	0.0323
8–9	0.38 (0.12–1.24)	0.1088	0.38 (0.14–1.07)	0.0681	0.87 (0.13–5.86)	0.8868	2.60 (0.96–7.03)	0.0605

All models adjusted for age, sex, CCI, education, marital status, living status, religious beliefs, income (NTD/month), and indwelling tube or catheter.

a DEPS scores: excluding missing (*n* = 13).

b ACP scores: excluding missing (*n* = 1).

## Discussion

This study provides new insight into how frailty shapes ACP processes among older adults receiving HHC. As frailty increased, decisional self-efficacy declined, yet readiness to engage in ACP was higher among those with moderate-to-severe frailty compared to mildly frail individuals. Importantly, greater engagement did not consistently translate into formal AD completion. Rather than viewing these findings as contradictory, they highlight a clinically meaningful divergence between perceived decision-making confidence and motivation to plan ahead. Recognizing this divergence is essential for identifying when and how to initiate supported ACP discussions in the context of progressive vulnerability.

Consistent with prior reports, self-efficacy for participation in medical decision-making declined as frailty increased (Whitehall et al. 2021). This pattern aligns with theoretical expectations that frailty, often compounded by multimorbidity, symptom burden, and functional dependence, can erode individuals’ confidence in navigating complex healthcare decisions. At the same time, accumulating experiences of clinical deterioration and repeated healthcare encounters may heighten awareness of future health uncertainties, thereby increasing openness to ACP discussions (Kinley and Flemming 2021). In contrast, mildly frail or relatively stable older adults may perceive ACP as less urgent or less relevant to their immediate circumstances (Wan et al. 2022).

The co-occurrence of declining decisional self-efficacy and heightened ACP readiness likely reflects 2 related but distinct constructs. Self-efficacy captures an individual’s confidence in actively leading medical discussions, whereas ACP engagement reflects recognition of the importance of articulating future care preferences. As physiological vulnerability progresses, these constructs may move in different directions: patients may feel less confident in managing complex medical conversations while simultaneously becoming more aware of the need for planning. This distinction underscores both the importance of tailoring ACP approaches to the patient’s stage of illness and decision-making capacity, and the proactive role of healthcare professionals in facilitating and supporting ACP initiatives among their clients by all means (Combes et al. 2021). Programs such as Singapore’s tiered national ACP framework, ranging from General ACP to Disease-Specific ACP and Preferred Plan of Care, illustrate how structured, stage-sensitive models may better align ACP engagement with evolving clinical trajectories (Ng et al. 2023).

However, the absence of increased AD completion should not be interpreted as a failure of ACP. Rather, it reflects a clinically important adaptation between psychological engagement and formal documentation. As frailty progresses, patients’ ability to engage in the complex process of ACP consultation, including completing documentation, might also decline. In this context, readiness may precede action, and engagement may not immediately translate into signed documents. Prior work has shown that self-efficacy in older adults is influenced by relational dynamics and prior patterns of health communication (Korpershoek et al. 2011; Remm et al. 2023), suggesting that reduced confidence does not necessarily imply disengagement. Therefore, ACP processes should prioritize structured, iterative, and values-based conversations that support participation before decisional confidence further diminishes. Approaches that emphasize relational dialogue over administrative completion, such as collaborative self-management models, may help bridge the gap between intention and formalization (Lorig et al. 1999; Thomas et al. 2017). Evaluating ACP success through alignment with patient values and quality of end-of-life engagement, rather than document acquisition alone, may provide a more meaningful measure of impact across the frailty spectrum (Malhotra and Ramakrishnan 2022; Malhotra 2023).

Several contextual factors may further explain why heightened readiness did not translate into document completion. Taiwan’s national health insurance does not routinely reimburse ACP consultations, and the requirement for out-of-pocket payment may deter formalization (He et al. 2021; Wu et al. 2023). Emotional avoidance of mortality, information overload during discussions, and decisional paralysis may also delay action despite expressed willingness (Lund et al. 2015). Sociocultural norms that frame end-of-life conversations as taboo (Lin et al. 2018), limited institutional training or standardized protocols (Lund et al. 2015; Blackwood et al. 2019), and family disagreement may further complicate the transition from intention to documentation. In addition, common misperceptions of ACP in Asia – that it is primarily financial planning, a legal document, or a conversation solely about death – may obscure its broader relational and values-based purpose (Martina et al. 2021a).

An additional nuance emerges at the highest levels of frailty. The odds of ACP engagement did not continue to rise linearly among individuals classified as very severely frail (CFS 8–9). In advanced frailty, profound multimorbidity, and cognitive impairment may constrain patients’ ability to further deepen ACP engagement, even if awareness of vulnerability remains high. At this stage, communication complexity, decisional fatigue, and reliance on surrogate participation may limit measurable increases in readiness. Moreover, the generally low rate of formal document completion in Taiwan may attenuate observable differences in this subgroup. This plateau effect suggests that ACP engagement may follow a curvilinear rather than strictly linear trajectory across the frailty spectrum.

Surrogate involvement in ACP discussions is common among individuals with advanced frailty or cognitive impairment (Golden et al. 2023). Although proxy-reported outcomes may not fully capture patients’ internal perspectives, evidence suggests reasonable alignment between patient and caregiver reports in domains such as quality of life (Takura et al. 2022), palliative care outcomes (Murtagh et al. 2019), and life-sustaining treatment decisions (Lin et al. 2020), with moderate concordance observed in cognitively impaired populations(Clapham et al. 2021; Kroenke et al. 2022). The ACP Engagement Survey has demonstrated strong internal consistency when completed by both individuals and surrogate decision-makers (Cronbach’s alpha: 0.90–0.91) (Van Scoy et al. 2019).

Within this context, it is ethically important to distinguish between substituted and supported decision-making. Substituted decision-making entails a surrogate making choices on behalf of an incapacitated individual based on presumed preferences, whereas supported decision-making provides assistance that enables individuals to express their own values and choices to the greatest extent possible. In progressive frailty, this distinction becomes particularly critical. The recent Consensus Definition of Advance Care Planning in Dementia from the European Association for Palliative Care emphasizes inclusivity in developing care plans for individuals with cognitive impairment (van der Steen et al. 2024). In our cohort, caregivers facilitated rather than dictated responses, reflecting clinical realities in many Asian settings where ACP is embedded within family-centered decision-making traditions (Lin et al. 2018; Chiang et al. 2021; Mori et al. 2025). These contextual considerations should inform the interpretation of the observed divergence between self-efficacy and ACP engagement.

The strength of our study resides in its utilization of a national cohort, employing stratified sampling across urban, suburban, and rural areas to examine the relationship between frailty, self-efficacy, and ACP in older adults. By recruiting patients from predefined regions, we aimed to minimize sampling bias, as the availability and utilization of healthcare services may exert an influence on self-efficacy and ACP. However, there are still several limitations. First, our findings are based on a national cohort in Taiwan, which limits the generalizability of our results to individuals from diverse ethnic backgrounds, cultural contexts, religious beliefs, and ethical ideals worldwide. Second, our results may not apply to older adults who are fully independent in their daily care. Third, the cross-sectional nature of this baseline analysis does not capture the dynamic, prospective fluctuations in self-efficacy and ACP engagement as frailty progresses or as health crises occur. Fourth, we did not assess our subjects’ baseline understanding of ACP. Given that greater knowledge of ACP is associated with an increased willingness to initiate ACP discussions (Ng et al. 2017), this omission may have led us to overlook factors influencing their engagement and responses. Finally, surrogate respondents may not accurately reflect the true decision-making self-efficacy and ACP readiness of patients. We recognize that patient-reported outcome measures enhance identification of unmet need, enabling healthcare professionals to address patients’ concerns more effectively, thereby reducing psychological distress and improving quality of life (Etkind et al. 2015). However, in real-world settings where cognitive impairment is prevalent, incorporating surrogate perspectives is essential to help healthcare professionals navigate the complexities of shared decision-making in ACP and respond appropriately. To address these challenges, we designed our study protocol based on the concept of supported decision-making (Davidson et al. 2015). This approach aligns with the clinical recommendations outlined in the *National Guidebook for Dementia Palliative Care in Taiwan*, developed by an expert panel (Chen 2024). To gain a more comprehensive understanding of the interplay between frailty, self-efficacy, and ACP during the transition, longitudinal follow-up studies and further analyses involving various cultural and geographical backgrounds are required.

## Conclusions

Frailty was linked to declining decisional self-efficacy yet greater readiness for ACP. This divergence indicates that growing vulnerability may increase motivation to plan ahead without ensuring formal documentation. Supporting meaningful patient engagement beyond document completion should be a priority in caring for frail older adults.

## Data Availability

The datasets generated and/or analyzed during the current study are not publicly available due to concerns regarding patient privacy and confidentiality but are available from the corresponding author on reasonable request.

## Table 1 Long description

The table compares participant characteristics across four Clinical Frailty Scale groups (scores 4–5, 6, 7, and 8–9) with group sizes 60, 83, 147, and 68, and reports P-values for between-group differences. Median age increases with frailty from 82 (Q1–Q3 75–87) at CFS 4–5 to 86 (81–92) at CFS 8–9 (P=0.0051), and the share aged 85+ rises from 38.3% to 61.8%. Comorbidity burden (Charlson Comorbidity Index) is higher in frailer groups: median 2 (1–3) at CFS 4–5 versus 3 (2–5) for CFS 6 through 8–9 (P=0.0081). Living arrangements shift strongly with frailty (P<0.0001): living only with family/friends drops from 70.0% (CFS 4–5) to 29.4% (CFS 8–9), while living with a carer and/or family/friend rises from 18.3% to 63.2%. Income distribution differs by frailty (P=0.0009), with the highest-income category (>60,000 NTD/month) increasing from 10.0% (CFS 4–5) to 29.4% (CFS 8–9), while the low-to-middle subsidy category decreases from 45.0% to 16.2%. Medical device use increases steeply with frailty: any indwelling tube/catheter is 8.3%, 22.9%, 49.0%, and 79.4% from CFS 4–5 to 8–9 (P<0.0001); urinary catheter and nasogastric tube show similar increases (both P<0.0001), while tracheostomy remains rare (0% to 5.9%; P=0.0550). Questionnaire respondents shift from patients to family/caregivers as frailty increases (patients: 51.7% at CFS 4–5 to 0% at CFS 8–9; P<0.0001). Sex, marital status, and religious beliefs do not show statistically significant differences across frailty groups (P=0.2645, 0.2087, and 0.6637). P-values reflect group comparisons using a nonparametric test and indicate association, not causation.

Navigate back to Table 1.

## Table 2 Long description

The table compares decision-making participation self-efficacy (DEPS) across Clinical Frailty Scale (CFS) groups: 4–5 (n=60), 6 (n=83), 7 (n=147), and 8–9 (n=68), with p-values for between-group differences. Median DEPS declines as frailty increases: 15.0 (Q1–Q3 9.0–23.0) at CFS 4–5, 11.0 (7.0–20.0) at CFS 6, 10.0 (6.0–20.0) at CFS 7 (5.4% missing), and 9.0 (5.0–17.0) at CFS 8–9 (7.4% missing), with a statistically significant overall difference (p=0.0353). For DEPS categories, the proportion scoring ≤9 rises with frailty (33.3% to 54.4%; p=0.0960), while scores >16 fall (41.7% to 23.5%). For five individual confidence items (Q1–Q5), “very to completely confident” generally decreases with higher CFS (e.g., Q1: 41.7% at CFS 4–5 vs 23.5% at CFS 8–9), but none of the item-level comparisons are statistically significant (p-values 0.1512 to 0.8843). Missing responses for items are reported only for CFS 7 (about 4–5%) and CFS 8–9 (7.4%). Results should be interpreted as nonparametric group comparisons using medians and interquartile ranges, and p-values reflect overall differences across all CFS groups rather than pairwise tests.

Navigate back to Table 2.

## Table 3 Long description

The table compares advance care planning (ACP) scores, readiness responses, and advance-directive completion across Clinical Frailty Scale (CFS) categories: 4–5 (n=60), 6 (n=83), 7 (n=147), and 8–9 (n=68). Median UCSF-ACP scores were 4.0 (IQR 4.0–6.0) for CFS 4–5, 5.0 (4.0–8.0) for CFS 6 (1 missing), 4.0 (4.0–8.0) for CFS 7, and 4.0 (4.0–8.0) for CFS 8–9, with no significant difference (p=0.2377). The proportion scoring exactly 4 ranged from 48.2% to 66.7% across groups (p=0.0724), while scores >6 ranged from 20.0% (CFS 4–5) to 39.8% (CFS 6). For readiness items, most participants in every frailty group selected “thinking about doing it to never thought” rather than “definitely planning/already done”: Q1 77.1%–85.0% (p=0.5708), Q2 89.2%–95.0% (p=0.6548), Q3 89.2%–93.3% (p=0.8269; 1 missing in CFS 6), and Q4 92.8%–98.3% (p=0.6007; 1 missing in CFS 6). Advance-directive completion was low in all groups—Yes: 10.0% (CFS 4–5), 9.6% (CFS 6), 4.8% (CFS 7), and 7.4% (CFS 8–9)—with no significant difference (p=0.4360; 1 missing in CFS 8–9). Overall, the data suggest little variation in ACP engagement by frailty level, and p-values indicate no statistically significant group differences.

Navigate back to Table 3.

## Table 4 Long description

Multinomial logistic regression results report adjusted odds ratios (ORs), 95% confidence intervals, and p-values for DEPS categories (10–16 and >16 vs ≤9) and ACP categories (5–6 and >6 vs 4) by frailty level. Frailty 4–5 is the reference group for all comparisons. For DEPS 10–16 vs ≤9, ORs decrease with higher frailty (6: 1.00; 7: 0.64; 8–9: 0.38) and none are statistically significant (p=0.9993, 0.3363, 0.1088). For DEPS >16 vs ≤9, ORs are also below 1 (6: 0.83; 7: 0.52; 8–9: 0.38) with no significant p-values (0.6714, 0.1180, 0.0681). For ACP 5–6 vs 4, ORs are near 1–2 (6: 1.98; 7: 1.81; 8–9: 0.87) and not significant (p=0.2723, 0.3231, 0.8868). For ACP >6 vs 4, higher frailty is associated with higher odds: frailty 6 OR 3.38 (95% CI 1.40–8.17, p=0.0069) and frailty 7 OR 2.52 (1.08–5.89, p=0.0323), while frailty 8–9 is elevated but not significant (OR 2.60, p=0.0605). Confidence intervals are wide for several estimates, so precision is limited even where point estimates suggest trends.

Navigate back to Table 4.

## References

[ref1] Arora NK, Weaver KE, Clayman ML, et al. (2009) Physicians’ decision-making style and psychosocial outcomes among cancer survivors. *Patient Education and Counseling* 77(3), 404–412. doi:10.1016/j.pec.2009.10.004 19892508 PMC3401045

[ref2] Blackwood DH, Walker D, Mythen MG, et al. (2019) Barriers to advance care planning with patients as perceived by nurses and other healthcare professionals: A systematic review. *Journal of Clinical Nursing* 28(23-24), 4276–4297. doi:10.1111/jocn.15049 31494997

[ref3] Boerner K, Carr D and Moorman S (2013) Family relationships and advance care planning: Do supportive and critical relations encourage or hinder planning? *The Journals of Gerontology Series B, Psychological Sciences and Social Sciences* 68(2), 246–256. doi:10.1093/geronb/gbs161 23286929 PMC3578259

[ref4] Charlson M, Szatrowski TP, Peterson J, et al. (1994) Validation of a combined comorbidity index. *Journal of Clinical Epidemiology* 47(11), 1245–1251. doi:10.1016/0895-4356(94)90129-5 7722560

[ref5] Chen PJ ed (2024) *National Guidebook for Dementia Palliative Care in Taiwan*. https://www.chimei.org.tw/main/cmh_department/59310/%E5%A4%B1%E6%99%BA%E5%AE%89%E5%AF%A7%E6%8C%87%E5%BC%95%E5%85%A8%E6%96%87.pdf.

[ref6] Chiang FM, Wang YW and Hsieh JG (2021) How acculturation influences attitudes about advance care planning and end-of-life care among Chinese living in Taiwan, Hong Kong, Singapore, and Australia. *Healthcare (Basel)* 9(11), 1477. doi:10.3390/healthcare9111477. 34828523 PMC8621689

[ref7] Chou YC, Tsou HH, Chan DD, et al. (2022) Validation of clinical frailty scale in Chinese translation. *BMC Geriatrics* 22(1), 604. doi:10.1186/s12877-022-03287-x. 35858829 PMC9298166

[ref8] Church S, Rogers E, Rockwood K, et al. (2020) A scoping review of the Clinical Frailty Scale. *BMC Geriatrics* 20(1), 393. doi:10.1186/s12877-020-01801-7. 33028215 PMC7540438

[ref9] Clapham S, Daveson BA, Allingham SF, et al. (2021) Patient-reported outcome measurement of symptom distress is feasible in most clinical scenarios in palliative care: An observational study involving routinely collected data. *International Journal for Quality in Health Care* 33(2), mzab075. doi:10.1093/intqhc/mzab075. 33909051 PMC8132729

[ref10] Combes S, Forbes G, Gillett K, et al. (2021) Development of a theory-based intervention to increase cognitively able frail elders’ engagement with advance care planning using the behaviour change wheel. *BMC Health Services Research* 21(1), 712. doi:10.1186/s12913-021-06548-4. 34284759 PMC8290869

[ref11] Crooms RC and Gelfman LP (2020) Palliative care and end-of-life considerations for the frail patient. *Anesthesia & Analgesia* 130(6), 1504–1515. doi:10.1213/ANE.0000000000004763. 32384340 PMC7536652

[ref12] Cybulski M, Cybulski L, Krajewska-Kulak E, et al. (2017) The level of emotion control, anxiety, and self-efficacy in the elderly in Bialystok, Poland. *Clin IntervAging* 12, 305–314. doi:10.2147/CIA.S128717. PMC5308481 28223788

[ref13] Davidson G, Kelly B, Macdonald G, et al. (2015) Supported decision making: A review of the international literature. *International Journal of Law and Psychiatry* 38, 61–67. doi:10.1016/j.ijlp.2015.01.008. 25676814

[ref14] de Vries K, Banister E, Dening KH, et al. (2019) Advance care planning for older people: The influence of ethnicity, religiosity, spirituality and health literacy. *Nursing Ethics* 26(7-8), 1946–1954. doi:10.1177/0969733019833130. 30943848

[ref15] Doba N, Tokuda Y, Saiki K, et al. (2016) Assessment of self-efficacy and its relationship with frailty in the elderly. *Internal Medicine* 55(19), 2785–2792. doi:10.2169/internalmedicine.55.6924. 27725537 PMC5088538

[ref16] Etkind SN, Daveson BA, Kwok W, et al. (2015) Capture, transfer, and feedback of patient-centered outcomes data in palliative care populations: Does it make a difference? A systematic review. *Journal of Pain and Symptom Management* 49(3), 611–624. doi:10.1016/j.jpainsymman.2014.07.010. 25135657

[ref17] Farley H (2020) Promoting self-efficacy in patients with chronic disease beyond traditional education: A literature review. *Nursing Open* 7(1), 30–41. doi:10.1002/nop2.382. 31871689 PMC6917929

[ref18] Golden SE, Rubim F, Thammana R, et al. (2023) Perspectives on advance care planning needs of persons with advanced dementia from their surrogates and clinicians. *PEC Innovation* 3, 100241. doi:10.1016/j.pecinn.2023.100241. 38076487 PMC10704328

[ref19] He YJ, Lin MH, Hsu JL, et al. (2021) Overview of the motivation of advance care planning: A study from a medical center in Taiwan. *International Journal of Environmental Research and Public Health* 18(2), 417. doi:10.3390/ijerph18020417. 33430302 PMC7825806

[ref20] Houben CHM, Spruit MA, Groenen MTJ, et al. (2014) Efficacy of advance care planning: A systematic review and meta-analysis. *Journal of the American Medical Directors Association* 15(7), 477–489. doi:10.1016/j.jamda.2014.01.008. 24598477

[ref21] Huang CH, Umegaki H, Kamitani H, et al. (2019) Change in quality of life and potentially associated factors in patients receiving home-based primary care: A prospective cohort study. *BMC Geriatrics* 19(1), 21. doi:10.1186/s12877-019-1040-3. 30678632 PMC6345012

[ref22] Kinley J and Flemming K (2021) Understanding what is important to older people living with frailty in relation to advance care planning. *International Journal of Palliative Nursing* 27(9), 471–480. doi:10.12968/ijpn.2021.27.9.471. 34846935

[ref23] Kojima G, Iliffe S and Walters K (2018) Frailty index as a predictor of mortality: A systematic review and meta-analysis. *Age Ageing* 47(2), 193–200. doi:10.1093/ageing/afx162. 29040347

[ref24] Korpershoek C, van der Bijl J and Hafsteinsdottir TB (2011) Self-efficacy and its influence on recovery of patients with stroke: A systematic review. *Journal of Advanced Nursing* 67(9), 1876–1894. doi:10.1111/j.1365-2648.2011.05659.x. 21645040

[ref25] Kroenke K, Stump TE and Monahan PO (2022) Agreement between older adult patient and caregiver proxy symptom reports. *Journal of Patient-Reported Outcomes* 6(1), 50. doi:10.1186/s41687-022-00457-8. 35567663 PMC9107556

[ref26] Krogseth M, Rostoft S, Benth JS, et al. (2021) Frailty among older patients receiving home care services. *Tidsskr den nor Laegeforening* 141(4). doi:10.4045/tidsskr.20.0688. 33685102

[ref27] Liao JY, Chen PJ, Wu YL, et al. (2020) HOme-based longitudinal investigation of the multidiSciplinary Team Integrated Care (HOLISTIC): Protocol of a prospective nationwide cohort study. *BMC Geriatrics* 20(1), 511. doi:10.1186/s12877-020-01920-1. 33246407 PMC7694342

[ref28] Lin CP, Cheng SY and Chen PJ (2018) Advance care planning for older people with cancer and its implications in Asia: highlighting the mental capacity and relational autonomy. *Geriatrics (Basel)* 3(3), 43. doi:10.3390/geriatrics3030043. 31011081 PMC6319225

[ref29] Lin CP, Peng JK, Chen PJ, et al. (2020) Preferences on the timing of initiating advance care planning and withdrawing life-sustaining treatment between terminally-Ill cancer patients and their main family caregivers: A prospective study. *International Journal of Environmental Research & Public Health* 17(21), 7954. doi:10.3390/ijerph17217954. 33138212 PMC7662916

[ref30] Lorig KR, Sobel DS, Stewart AL, et al. (1999) Evidence suggesting that a chronic disease self-management program can improve health status while reducing hospitalization: A randomized trial. *Medical Care* 37(1), 5–14. doi:10.1097/00005650-199901000-00003. 10413387

[ref31] Lund S, Richardson A and May C (2015) Barriers to advance care planning at the end of life: An explanatory systematic review of implementation studies. *PLoS One* 10(2), e0116629. doi:10.1371/journal.pone.0116629. 25679395 PMC4334528

[ref32] Malhotra C (2023) Advance care planning: It is time to rethink our goals. *Journal of the American Geriatrics Society* 71(12), 3963–3966. doi:10.1111/jgs.18511. 37522615

[ref33] Malhotra C and Ramakrishnan C (2022) Complexity of implementing a nationwide advance care planning program: Results from a qualitative evaluation. *Age Ageing* 51(10), afac224. doi:10.1093/ageing/afac224. 36273345

[ref34] Martina D, Geerse OP, Lin CP, et al. (2021a) Asian patients’ perspectives on advance care planning: A mixed-method systematic review and conceptual framework. *Palliative Medicine* 35(10), 1776–1792. doi:10.1177/02692163211042530. 34488509 PMC8637390

[ref35] Martina D, Lin CP, Kristanti MS, et al. (2021b) Advance care planning in Asia: A systematic narrative review of healthcare professionals’ knowledge, attitude, and experience. *Journal of the American Medical Directors Association* 22(2), 349e341–349e328. doi:10.1016/j.jamda.2020.12.018. 33421371

[ref36] Maruta M, Makizako H, Ikeda Y, et al. (2022) Characteristics of meaningful activities in community-dwelling Japanese older adults with pre-frailty and frailty. *Archives of Gerontology and Geriatrics* 99, 104616. doi:10.1016/j.archger.2021.104616. 35016133

[ref37] Mori M, Chan HYL, Lin CP, et al. (2025) Definition and recommendations of advance care planning: A Delphi study in five Asian sectors. *Palliative Medicine* 39(1), 99–112. doi:10.1177/02692163241284088. 39390784 PMC11673296

[ref38] Murtagh FE, Ramsenthaler C, Firth A, et al. (2019) A brief, patient- and proxy-reported outcome measure in advanced illness: Validity, reliability and responsiveness of the integrated palliative care outcome scale (IPOS). *Palliative Medicine* 33(8), 1045–1057. doi:10.1177/0269216319854264. 31185804 PMC6691591

[ref39] Ng QX, Kuah TZ, Loo GJ, et al. (2017) Awareness and attitudes of community-dwelling individuals in singapore towards participating in advance care planning. *Annals of the Academy of Medicine, Singapore* 46(3), 84–90. doi:10.47102/annals-acadmedsg.v46n3p84 28417132

[ref40] Ng R, Lip Hoe K, Lim J, et al. (2023) Advance care planning in Singapore: The genesis and evolution of a national programme. *Z Evid Fortbild Qual Gesundhwes* 180, 99–102. doi:10.1016/j.zefq.2023.05.018. 37407336

[ref41] Proietti M and Cesari M (2020) Frailty: What is it? *Advances in Experimental Medicine and Biology* 1216, 1–7. doi:10.1007/978-3-030-33330-0_1. 31894541

[ref42] Remm S, Halcomb E, Hatcher D, et al. (2023) Understanding relationships between general self-efficacy and the healthy ageing of older people: An integrative review. *Journal of Clinical Nursing* 32(9-10), 1587–1598. doi:10.1111/jocn.16104. 34716612

[ref43] Ritchie CS and Leff B (2018) Population health and tailored medical care in the home: The roles of home-based primary care and home-based palliative care. *Journal of Pain and Symptom Management* 55(3), 1041–1046. doi:10.1016/j.jpainsymman.2017.10.003. 29031914

[ref44] Ritchie CS, Leff B, Garrigues SK, et al. (2018) A quality of care framework for home-based medical care. *Journal of the American Medical Directors Association* 19(10), 818–823. doi:10.1016/j.jamda.2018.05.020. 30056010 PMC6392035

[ref45] Soones T, Federman A, Leff B, et al. (2017) Two-year mortality in homebound older adults: An analysis of the national health and aging trends study. *Journal of the American Geriatrics Society* 65(1), 123–129. doi:10.1111/jgs.14467. 27641001 PMC5258674

[ref46] Sudore RL, Heyland DK, Barnes DE, et al. (2017) Measuring advance care planning: Optimizing the advance care planning engagement survey. *Journal of Pain and Symptom Management* 53(4), 669–681e668. doi:10.1016/j.jpainsymman.2016.10.367. 28042072 PMC5730058

[ref47] Sudore RL, Lum HD, You JJ, et al. (2017) Defining advance care planning for adults: A consensus definition from a multidisciplinary Delphi panel. *Journal of Pain and Symptom Management* 53(5), 821–832.e821. doi:10.1016/j.jpainsymman.2016.12.331. 28062339 PMC5728651

[ref48] Takura T, Koike T, Matsuo Y, et al. (2022) Proxy responses regarding quality of life of patients with terminal lung cancer: Preliminary results from a prospective observational study. *BMJ Open* 12(2), e048232. doi:10.1136/bmjopen-2020-048232. PMC8883223 35210333

[ref49] Thomas PA, Liu H and Umberson D (2017) Family relationships and well-being. *Innovation in Aging* 1(3), igx025. doi:10.1093/geroni/igx025. 29795792 PMC5954612

[ref50] van der Steen JT, Nakanishi M, den Block L V, et al. and European Association for Palliative C (2024) Consensus definition of advance care planning in dementia: a 33-country Delphi study. *Alzheimer’s & Dementia* 20(2), 1309–1320. doi:10.1002/alz.13526. PMC10916978 37985444

[ref51] Van Scoy LJ, Day AG, Howard M, et al. (2019) Adaptation and preliminary validation of the advance care planning engagement survey for surrogate decision makers. *Journal of Pain and Symptom Management* 57(5), 980–988e989. doi:10.1016/j.jpainsymman.2019.01.008. 30684633 PMC6857702

[ref52] Wan Z, Chan HYL, Chiu PKC, et al. (2022) Experiences of older adults with frailty not completing an advance directive: A qualitative study of ACP conversations. *International Journal of Environmental Research & Public Health* 19(9), 5358. doi:10.3390/ijerph19095358 35564755 PMC9104599

[ref53] Wei FC, Hsu CK, Wu YL, et al. (2022) Reliability and validity of the traditional chinese version of the advance care planning engagement survey: A pilot evaluation in Taiwanese outpatients. *Journal of Palliative Care* 37(3), 273–279. doi:10.1177/08258597211051208. 34787527 PMC9344488

[ref54] Whitehall L, Rush R, Gorska S, et al. (2021) The general self-efficacy of older adults receiving care: A systematic review and meta-analysis. *Gerontologist* 61(6), e302–e317. doi:10.1093/geront/gnaa036. 32373938 PMC8361502

[ref55] Wu YL, Yang CY, Lin TW, et al. (2023) Factors impacting advance decision making and health care agent appointment among taiwanese urban residents after the passage of patient right to autonomy act. *Healthcare (Basel)* 11(10), 1478. doi:10.3390/healthcare11101478. 37239764 PMC10217947

