# Frailty and the confidence to plan ahead: Decision-making self-efficacy and advance care planning among older adults receiving home healthcare– CORRIGENDUM

**DOI:** 10.1017/S1478951526103022

**Published:** 2026-07-17

**Authors:** Chi Hsien Huang, Cheng-pei Lin, Kelly Yen-chih Chen, Jung-Yu Liao, Shao-Yi Cheng, Wei-Zhe Tseng, Hiroyuki Umegaki, Hsien-Cheng Chang, Chia-Ming Li, Wen-Jung Sun, Hung-Yi Chiou, Sang-Ju Yu, Chao A. Hsiung, Ping-Jen Chen

The authors apologise for the omission of two grant numbers from the Funding section in the published article. The Funding section has now been corrected in the article and now reads as follows:

Funding. This work was supported by the National Health Research Institutes of Taiwan (PH-112-GP-06), National Science and Technology Council of Taiwan (112–2410-H-037-005-MY3 and 112–2314-B-650-001-MY3), E-Da Hospital (EDPJ114023, EDAHJ114008 and EDAHS114019), and a grant from the Kaohsiung Medical University Hospital (KMUH114-4R83). The funders had no role in study design, methods, data collection, analysis, and preparation of the manuscript.
